# The Chloroplast Min System Functions Differentially in Two Specific Nongreen Plastids in *Arabidopsis thaliana*

**DOI:** 10.1371/journal.pone.0071190

**Published:** 2013-07-30

**Authors:** Peng Wang, Jie Zhang, Jianbin Su, Peng Wang, Jun Liu, Bing Liu, Dongru Feng, Jinfa Wang, Hongbin Wang

**Affiliations:** Guangdong Key Laboratory of Plant Resources and State Key Laboratory of Biocontrol, School of Life Sciences, Sun Yat-sen University, Guangzhou, People’s Republic of China; Iowa State University, United States of America

## Abstract

The nongreen plastids, such as etioplasts, chromoplasts, etc., as well as chloroplasts, are all derived from proplastids in the meristem. To date, the Min system members in plants have been identified as regulators of FtsZ-ring placement, which are essential for the symmetrical division of chloroplasts. However, the regulation of FtsZ-ring placement in nongreen plastids is poorly understood. In this study, we investigated the division site placement of nongreen plastids by examining the etioplasts as representative in Arabidopsis Min system mutants. Surprisingly, the shape and number of etioplasts in cotyledons of *arc3*, *arc11* and *mcd1* mutants were similar to that observed in wild-type plants, whereas *arc12* and *parc6* mutants exhibited enlarged etioplasts that were reduced in number. In order to examine nongreen plastids in true leaves, we silenced the *ALB3* gene in these Min system mutant backgrounds to produce immature chloroplasts without the thylakoidal network using virus induced gene silencing (VIGS). Interestingly, consistent with our observations in etioplasts, enlarged and fewer nongreen plastids were only detected in leaves of *parc6* (VIGS-ALB3) and *arc12* (VIGS-ALB3) plants. Further, the FtsZ-ring assembled properly at the midpoint in nongreen plastids of *arc3*, *arc11* and *mcd1* (VIGS-ALB3) plants, but organized into multiple rings in *parc6* (VIGS-ALB3) and presented fragmented filaments in *arc12* (VIGS-ALB3) plants, suggesting that division site placement in nongreen plastids requires fewer components of the plant Min system. Taken together, these results suggest that division site placement in nongreen plastids is different from that in chloroplasts.

## Introduction

It is widely accepted that chloroplasts arose from cyanobacteria more than one billion years ago through the establishment of endosymbiosis with their hosts [1], [2], [3]. Chloroplasts are essential for photosynthesis, which is the process of converting solar energy to chemical energy, and they also provide the site for nitrogen and sulfur assimilation, as well as biosynthesis of amino acids, lipids, hormones, sugars and starch in plants [4].

To maximize the photosynthetic efficiency of a living cell, an appropriate chloroplast population within cells needs to be finely regulated. In Arabidopsis, chloroplasts are not produced de novo, but replicate mainly by binary fission from proplastids in the meristem [1], [5]. The multi-subunit division protein complex is composed of proteins derived from the original endosymbiont genome as well as proteins recruited from the eukaryotic host cell during evolution [6]. Similar to its cyanobacterial ancestor, chloroplast division requires the FtsZ protein, which is a tubulin-like cytoskeletal GTPase that can form a ring-shaped structure (the FtsZ-ring) in the middle of the chloroplast, which is the first and very important event before the onset of chloroplast constriction for symmetrical division [6], [7], [8], [9].

In *E.coli*, the placement of the FtsZ-ring is dynamically regulated by the Min (*Mini*cell) system, which is composed of the direct FtsZ assembly inhibitor MinC, and the MinD and MinE proteins [10], [11], [12]. They oscillate at the cell in a pole-to-pole manner; thus, the FtsZ assembly is inhibited most at the poles and only restricted to the midcell [13]. In plants, homologs of the bacterial MinD and MinE proteins have been identified by reverse genetics, and mutant analysis showed that both MinD1 and MinE1 were required for chloroplast division site placement [14], [15], [16]. MinC has been lost from most eukaryotic lineages, and it is proposed that ARC3 fulfills the function of MinC in the Viridiplantae [17], [18]. Because ARC3 overexpression inhibits chloroplast division and it interacts with MinD1, MinE1, FtsZ1 [17] and PARC6 [19], it seems to inhibit the assembly of FtsZ1 directly. PARC6 [19], also known as CDP1 [20], which appears to be present only in vascular plants, was shown to promote FtsZ filament formation and function in FtsZ-ring placement. MULTIPLE CHLOROPLASTS DIVISION SITE 1 (MCD1), a protein unique to land plants, was shown to interact with MinD1, localizing at the division site and punctuate structures dispersed on the inner envelope, to regulate division site placement [21]. It appears that a functional protein complex known as the Min system, consisting of MinD1, MinE1, ARC3, PARC6/CDP1 and MCD1, is required to regulate FtsZ-ring placement in chloroplasts. Most recently, the mechanosensitive ion channel proteins MSL2 and MSL3 were also reported to affect chloroplast division site placement [22], [23]. Genetic studies have shown that MSL2 and MSL3 regulate FtsZ-ring formation via MinD1, MinE1 and ARC3. However, MSL2 and MSL3 do not interact with MinD1, MinE1, ARC3 or PARC6 directly [23]. Thus, it remains to be determined whether MSL2 and MSL3 are components of the plant Min system. Following division site placement, several division proteins begin to assemble from the inside to the outside of chloroplasts in the order of FtsZ-ring, inner plastid division (PD) ring, outer PD ring and the DPR5B ring [6], [24], [25].

During evolution, proplastids differentiated into different types of plastids in vascular plants; the chloroplasts, chromoplasts, leucoplasts, amyloplasts, etc. [26]. Most of our knowledge of plastid division comes from studying chloroplast division in leaf mesophyll cells. Because nongreen plastids are always smaller and more irregularly shaped than chloroplasts, the division mechanism in nongreen plastids is poorly understood. It has recently been proposed that the division mechanism of nongreen plastids is similar but not identical with that in chloroplasts [25], [27]. For example, no proplastid division defect was observed in the Arabidopsis *arc5* mutant, which displays a severe chloroplast division defect [28]. Although a difference was observed between chloroplast and nongreen plastid division, whether the mechanism of division site placement is different among different types of plastids remains to be determined.

In this study we examined the morphology of nongreen plastids and their FtsZ-ring placement in Min system mutants of Arabidopsis. Consistent with the situation in chloroplasts, we found that in nongreen plastids, *ARC12* and *PARC6* were also required to determine division site placement. Surprisingly, the morphology and FtsZ-ring placement of nongreen plastids in *arc3*, *arc11* and *mcd1* mutants were almost identical with that in wild-type, suggesting that division site placement in nongreen plastids requires fewer components of the plant Min system. Our study not only demonstrates that division site placement is differently regulated between chloroplasts and nongreen plastids, but also provides a valuable strategy to study nongreen plastid division in true leaves.

## Materials and Methods

### Plant Materials and Growth Conditions

The *Arabidopsis thaliana* ecotypes Columbia (Col-0), Landsberg erecta (Ler) and all the Min system mutants were grown in soil at 22°C in a growth chamber under a 16 h light/8 h dark photoperiod with 60% humidity. For etiolated seedling analysis, seeds were surface-sterilized using 70% ethanol for 1 min followed by 20% bleach for 15 min and sown on half-strength Murashige and Skoog (MS) plates. After vernalization for 3 days at 4°C, the plates were placed in the dark for 6 days at 22°C and illuminated under 110 µmol photons m^−2^ s^−1^ for 12 h and 36 h respectively. The mutants used in this study were obtained from the ABRC as follows: *arc3* (At1g75010, CS264), *arc11* (At5g24020, CS281), *arc12* (At1g69390, CS16472), *mcd1* (At1g20830, CS65539) and *parc6* (At3g19180, CS860043).

### Quantitative Real-time PCR

For quantitative real-time PCR (qRT-PCR) analyses, total RNA was extracted using the Plant RNeasy Mini Kit (Omega). Five micrograms total RNA was used for cDNA synthesis using the PrimeScript® RT reagent Kit (Takara) according to the manufacturer’s instructions. qRT-PCR was performed using SYBR® Premix Ex TaqTM (Takara) and amplification was monitored in real-time on the LightCycler480 (Roche). *UBQ4* was amplified as internal control, and gene copy number was normalized to *UBQ4*. The primers used for qRT-PCR analysis are as follows: *ARC3*, 5′-ACTGTTCATGAGCCTGATCTGG-3′ and 5′-CAGAAAAAATGTTGCCTTTGT-TG-3′; *ARC11*, 5′-CTTGTTCTGAATAAGCCTCCTACG-3′ and 5′-GCCACGTTTC-TTAGGTTCTTCC-3′; *ARC12*, 5′-CTCTGCGACCTTAGTATCTCCTTATC-3′ and 5′-ATTGCTTATGAATCCCGTGAAAT-3′; *PARC6*, 5′-GATCTTGGGGAAAGAG-TCGAGA-3′ and 5′-TGGAGAACAACCCCTTGTGTCT-3′; *MCD1*, 5′-ACATTCCC-CATGATTCCGAGTA-3′ and 5′-CCCCAATCCAAATGCTGTTC-3′; *FtsZ1*, 5′-CA-GATGATGTTTTACGCCAAGG-3′ and 5′-AGAAACACCTACCCCGAGCA-3′; *UBQ4*, 5′-CTGTTCACGGAACCCAATT-3′ and 5′-GGAAAAAGGTCTGACCGAC-A-3′.

### Microscopy

For cotyledons observation, etioplasts and chloroplasts were detected directly by tapping the cotyledons on the slides to release the cells. And for VIGS plants, protoplasts were prepared for observation the morphology of mature and immature chloroplasts. Leaves of 4-week-old infected plants were cut into strips and digested with enzyme solution (1.5% cellulase R-10, 0.4% macerozyme R-10, 0.4 M mannitol, 20 mM MES [pH 5.7], 10 mM CaCl_2_ and 0.1% BSA) for 2 h in the dark, and protoplasts were harvested by centrifugation and washed once with W5 solution (154 mM NaCl, 125 mM CaCl_2_, 5 mM KCl and 2 mM MES [pH 5.7]). The protoplasts were observed using a laser scanning confocal microscope (Leica TCS5 SP5 AOBS) and visualized with the Leica Microsystem LAS AF software. Transmission electron microscopy (TEM) was performed as described [29]. Leaves were cut into 2×3 mm^2^ pieces and fixed in 2% para-formaldehyde, 2.5% glutaraldehyde in 0.1 M phosphate buffer, pH 7.4, overnight at 4°C. Images were captured with a JEM1400 TEM (JEOL) at 120 kV according to the manufacturer’s instructions.

### Virus Induced Gene Silencing of *ALB3*

The VIGS vectors pTRV1 and pTRV2 are based on tobacco rattle virus [30]. A fragment of the *ALB3* open reading frame was amplified using primers 5′-GCATCTAGAGATTAAACCGTCGTCATC-3′ and 5′-CATCTCGAGTACTTGTT-GGGCGGTA-3′ and inserted into the pTRV2 vector. The pTRV2-GFP vector was used as negative control. pTRV1 or pTRV2 and its derivatives were transformed into *Agrobacterium tumefaciens* strain GV3101. The VIGS assay was carried out as described [31].

### Chlorophyll Fluorescence and Pigment Analysis

Chlorophyll was extracted from whole leaves with 80% acetone in 2.5 mM Hepes-KOH (pH 7.5), and the chlorophyll content was determined as described by Wellburn [47]. Chlorophyll fluorescence parameters were determined using a MAXI-IMAGING PAM chlorophyll fluorometer (Walz, Effe ltrich, Germany). Minimum fluorescence (*F_o_*) and maximum fluorescence (*F_m_*) yields of dark adapted leaves were measured at <1 µmol photons m^−2^ s^−1^ and 2700 µmol photons m^−2^ s^−1^ at 1 Hz. Maximum quantum yield of PSII (*F_v_/F_m_)* was calculated using (*F_m_−F_o_*)/*F_m_*.

### Immunofluorescence Analysis of FtsZ

Chloroplast preparation and immunofluorescence detection were carried out as previously described [23], [32] with slight modifications. The protoplasts were prepared as described above. Then the chloroplasts were released by tapping the protoplasts firmly and were fixed to the slides treated with poly-L-lysine. The slides were blocked with 3% BSA in PBS for 20 min, anti-FtsZ2-1 antibody (antipeptide antibodies against AtFtsZ2-1 were raised in rabbits and affinity-purified as described [9]) was applied at a 1∶600 dilution and incubated overnight at room temperature, and the slides were then incubated with Alex 488 conjugated goat-anti-rabbit secondary antibody (1∶400) for 1 h. Alex 488 fluorescence was observed with a laser scanning confocal microscope (Leica TCS5 SP5 AOBS) and visualized with the Leica Microsystem LAS AF software.

## Results

### Mutation of Min System Members PARC6 and ARC12 Affects Etioplast Division

**Figure 1:**
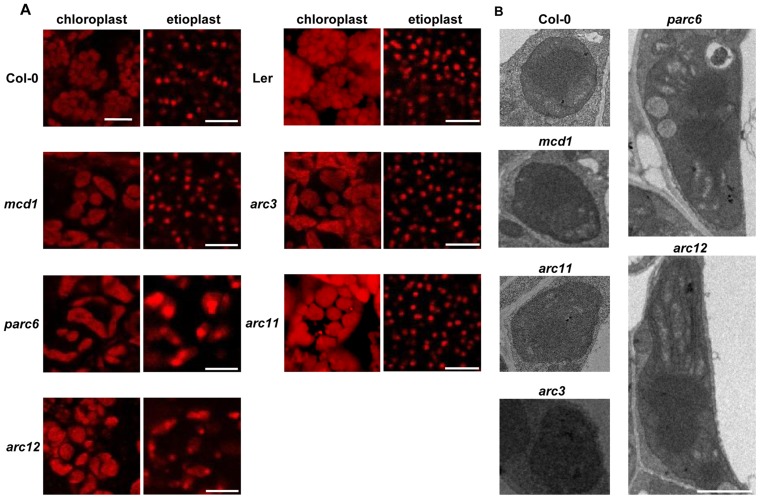
Morphology of etioplasts and chloroplasts in Arabidopsis wild-type and Min system mutants. (**A**) Chlorophyll autofluorescence images of etioplasts and chloroplasts were captured from cotyledons of 6-day-old etiolated seedlings and green seedlings grown under a 16 h light/8 h dark photoperiod with an irradiance of 110 µmol photons m^−2^ s^−1^ by confocal laser microscopy (CLSM). Scale bars = 10 µm. (**B**) Transmission electron microscopy (TEM) micrographs of etioplast ultrastructure in Col-0 and Min system mutants. Scale bars = 2 µm.

Previous studies have shown that division in nongreen plastids utilizes division machinery similar but not identical to that in chloroplast [25], [27]; thus, we speculated whether the Min system proteins affected the FtsZ-ring placement in nongreen plastids (etioplasts as representative) similar to what is seen in chloroplasts. To address this question, we first analysed the expression profile of the Min system genes by quantitative real-time PCR (qRT-PCR) in the cotyledon of the etiolated seedlings and the seedlings illuminated for 12 h and 36 h, in which condition the etioplasts have developed into mature chloroplasts. Expression levels of the Min genes were all up-regulated for 3–5 folds (Figure S1), so whether all of them play similar roles in the etioplast division as in the chloroplast? To test this hypothesis, etioplasts from 6-day-old etiolated seedlings cotyledons of the Arabidopsis Min system mutants and the wild-type were examined by confocal laser microscopy (CLSM). Surprisingly, we found that not all the mutants exhibited etioplast division defects as observed in chloroplasts of these mutants. Huge and abnormal etioplasts were observed in etiolated seedlings of *parc6* and *arc12* mutants; however, the etioplasts of *arc3*, *arc11* and *mcd1* mutants were normal in number and regular in size, similar to those of the wild-type (Figure 1A). In contrast, for the green cotyledons, all the five mutants exhibited abnormal chloroplasts similar to the phenotype of mature leaves as previous study showed (Figure 1A).

To further confirm this result, we examined the ultrastructure of etioplasts by transmission electron microscopy (TEM). Etioplasts in the wild-type were about 1–2 µm with a prolamellar body (PLB) ([33], [34], [35], Figure 1B), which is the signature of etioplasts [3], but in the *parc6* and *arc12* mutants, the etioplasts were enlarged (6–8 µm) and were irregular (Figure 1B). Interestingly, there was not one PLB structure visible in the *parc6* and *arc12* etioplasts (Figure 1B), which seemed to indicate that etioplast division was restrained in *parc6* and *arc12*. In contrast, both the size and shape of the etioplasts in the other three mutants (*arc3*, *arc11* and *mcd1)* were similar with that observed in wild-type (Figure 1B).

It has been reported that a deficiency of Min system proteins leads to enlarged and abnormal shaped chloroplasts [25], [36], whereas we found that only deletion of PARC6 and ARC12 affected etioplast division (Figure 1B). These results suggest that PARC6 and ARC12 are required for etioplast division, but deletion of ARC3, ARC11 and MCD1 do not display defective phenotype of etioplast division.

### Mutation of Min System Members PARC6 and ARC12 also Affects Nongreen Plastid Division in True Leaves

**Figure 2:**
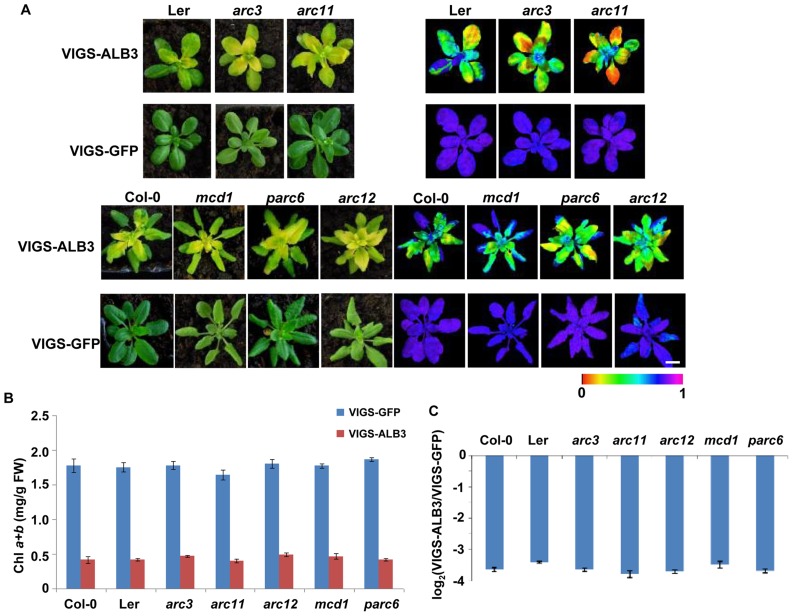
Characterization of the Arabidopsis VIGS plants in wild-type and Min system mutants. (**A**) Phenotypes of the VIGS-GFP and VIGS-ALB3 silenced plants. Images of representative plants taken 3 weeks after infection are shown on the left. Images of chlorophyll fluorescence parameters *F_v_/F_m_* of the infected plants captured by a MAXI-IMAGING PAM chlorophyll fluorometer are shown on the right. Scale bars = 1 cm. (**B**) Chlorophyll contents of the VIGS-GFP and VIGS-ALB3 plants, FW, fresh weight. (**C**) Relative level of silencing of *ALB3* mRNA in VIGS-ALB3 plants as analyzed by qRT-PCR. Data are given as means ± SD of three biological replicates.

Further, we also wanted to test whether PARC6 and ARC12 affected nongreen plastid division in true leaves (as opposed to cotyledons). To address this question, we silenced the *ALBINO3* (*ALB3*) gene in Min system mutants using virus induced gene silencing (VIGS) [30], [37]. Previous studies showed that chloroplasts in *alb3* mutants were far less organized with very few thylakoid membranes [38], [39], [40]. Thus, we silenced the *ALB3* gene in Min system mutant backgrounds to generate nongreen plastids in expanding true leaves. A 1000-bp cDNA fragment of *ALB3* was inserted into the VIGS vector pTRV2, which was then used to infect 12-day-old mutants and wild-type seedlings. Approximately two weeks later, the newly emerged leaves of VIGS-ALB3 plants were yellow or slightly albino, while the VIGS-GFP plants (a negative control) remained green (Figure 2A). Photosynthetic capacity (*F_v_/F_m_*) (Figure 2A) and chlorophyll content of VIGS-ALB3 plants were both severely reduced compared to the negative control (Figure 2B). qRT-PCR revealed that *ALB3* expression was reduced by approximately 90% in the VIGS-ALB3 plants (Figure 2C). In parallel, the expression of five Min system genes in VIGS-GFP and VIGS-ALB3 plants were comparable, indicating that their expression was not affected by silencing *ALB3* (Figure S2). Taken together, these results demonstrated that VIGS-mediated silencing of *ALB3* could result nongreen plastids in true leaves.

We next examined the morphology of the nongreen plastids in these VIGS plants. To this end, we isolated protoplasts from the VIGS plants three weeks after infection and checked these nongreen plastids by CLSM and TEM. Consistent with etioplasts, the nongreen plastids of *parc6* (VIGS-ALB3) and *arc12* (VIGS-ALB3) were fewer in number and larger in size than in the wild-type (VIGS-ALB3) (Figure 2A, Figure S3). In contrast, the other three mutants infected with TRV-ALB3 displayed normal nongreen plastids similar to wild-type (VIGS-ALB3) (Figure 2A, Figure S3). As expected, for the VIGS-GFP plants, all five mutants exhibited abnormal chloroplasts relative to wild-type (VIGS-GFP) (Figure 2A).

**Figure 3:**
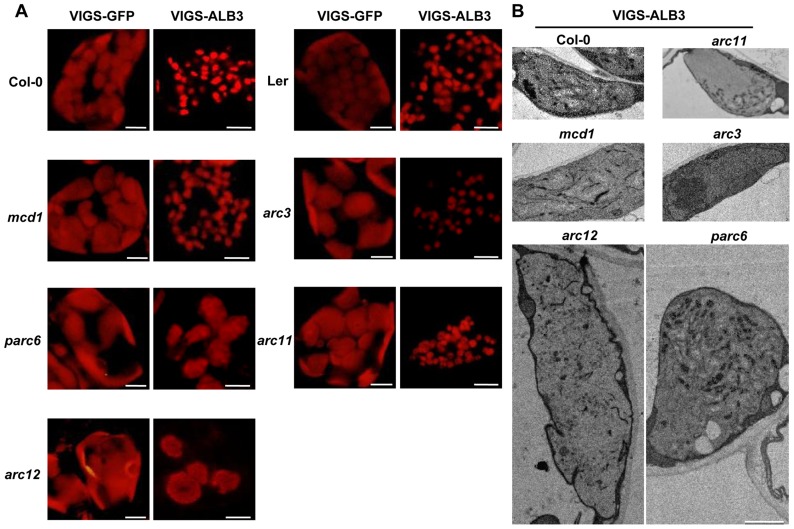
Morphology of nongreen plastids in Arabidopsis Min system mutant plants infected with TRV-GFP or TRV-ALB3. All plants were infected with TRV-GFP or TRV-ALB3 at 12 days old. Nongreen plastids were observed 3 weeks after infection. (**A**) Confocal chlorophyll autofluorescence images of nongreen plastids were captured from leaf mesophyll cells. Scale bars = 10 µm. (**B**) Transmission electron microscopy (TEM) micrographs of nongreen plastid ultrastructure in VIGS-ALB3 plants in wild-type and Min system mutants. Scale bars = 2 µm.

Next we investigated the ultrastructure of the nongreen plastids in wild-type and Min system mutants (VIGS-ALB3) by TEM. Consistent with the *alb3* mutants, the thylakoids in these nongreen plastids had few lamella present in the stroma (Figure 3B). Supporting these results, the sizes of the nongreen plastids in *arc12* (VIGS-ALB3) and *parc6* (VIGS-ALB3) were much larger than those in the other three mutants and wild-type (VIGS-ALB3) (Figure 2A, Figure 3B). Taken together, our results further demonstrated that deletion of ARC3, ARC11 and MCD1 do not affect the number and size of nongreen plastids, but PARC6 and ARC12 are necessary for the division of nongreen plastids in true leaves.

### Mutation of PARC6 and ARC12 Affects FtsZ-ring Placement and Assembly in Nongreen Plastids

**Figure 4:**
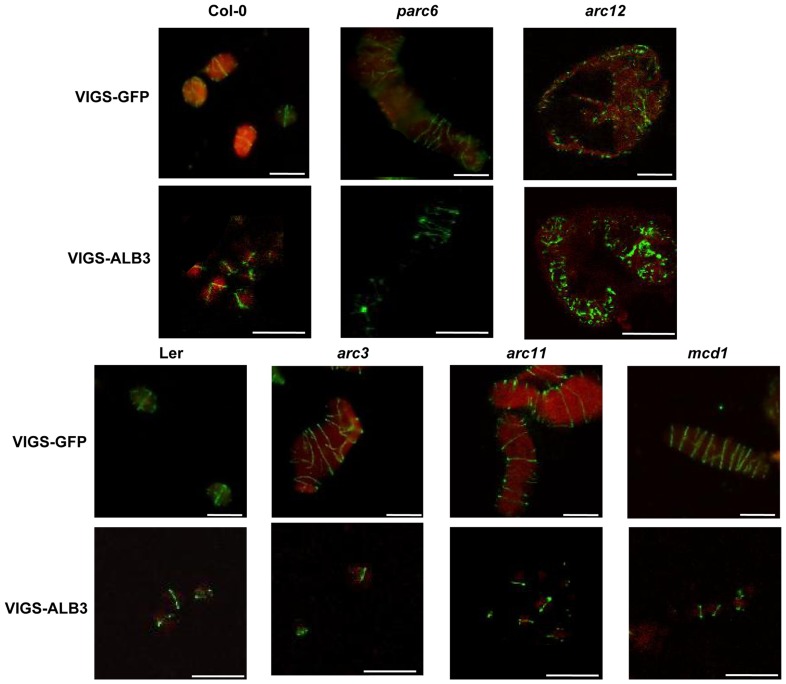
Immunofluorescence analysis of the FtsZ-ring in VIGS plants. Immunofluorescence images of the FtsZ2-1 ring of chloroplasts in VIGS-GFP plants and nongreen plastids in VIGS-ALB3 plants two weeks after infection. Chloroplasts are indicated by chlorophyll autofluorescence (red). Scale bars = 10 µm.

The Min system has been shown to determine placement of the FtsZ-ring at the midpoint of the chloroplast to yield two daughter chloroplasts of equal size. *arc3*, *arc11* and *mcd1* mutants displayed multiple FtsZ-rings throughout the enlarged chloroplasts, while the *parc6* mutant presented long FtsZ filaments spiraling the chloroplasts and the *arc12* mutant had numerous short and inordinate FtsZ filaments [16], [17], [19], [20], [21], [41]. We next intended to examine the effects of these Min system members on the assembly and placement of the FtsZ-ring in nongreen plastids. Using immunofluorescence, we examined the organization of FtsZ in the VIGS-ALB3 and VIGS-GFP plants. Multiple FtsZ-rings were observed in the *arc3* (VIGS-GFP), *arc11* (VIGS-GFP) and *mcd1* (VIGS-GFP) plants (Figure 4). In the corresponding VIGS-ALB3 plants, however, FtsZ proteins assembled into only one FtsZ-ring at the midpoint of the nongreen plastids similar to wild-type (VIGS-ALB3) (Figure 4). This indicates that FtsZ proteins can assemble and be placed properly in the absence of the ARC3, ARC11 or MCD1 proteins within nongreen plastids.

The irregularity of plastid division in the *parc6* (VIGS-ALB3) and *arc12* (VIGS-ALB3) plants suggested that the FtsZ proteins might be disorganized in them. As expected, we observed multiple FtsZ-rings in *parc6* (VIGS-ALB3) nongreen plastids and disordered filaments of FtsZ proteins in *arc12* (VIGS-ALB3) nongreen plastids (Figure 3). Together, our results suggest that deletion of ARC3, ARC11 and MCD1 do not influence assembly and placement of the FtsZ-ring in nongreen plastids, whereas PARC6 and ARC12 are required for division site placement in both chloroplasts and nongreen plastids.

## Discussion

Previous results obtained in Arabidopsis [19], [42], [43] showed that before constriction of the chloroplast, plastid division proteins form several plastid division (PD) rings in the order of FtsZ, inner PD, outer PD, and DRP5B rings. Regulation of FtsZ-ring placement in chloroplasts seems to be more complicated than it is in *E. coli*. Apart from ARC11 and ARC12, which are homologs of bacterial MinD and MinE [13], [24], the host derived proteins ARC3, PARC6 and MCD1 are also essential components required for the regulation of chloroplast FtsZ-ring placement. Recently, the mechanosensitive ion channel proteins MSL2 and MSL3 [22], [23] were also reported to affect chloroplast division site placement. Despite these excellent advances in clarifying the mechanism of chloroplast division, the machinery governing the division in nongreen plastids remains poorly understood. This is mostly because the nongreen plastids are colorless and much smaller than chloroplasts, which makes them difficult to observe and study [27].

Findings in the *arc* mutants reveal that both *arc5* [28] and *arc6* [44] mutations result in severe chloroplast division defects. However, proplastid division defects are observed in *arc6* [44] but not *arc5* mutants [28], [45], indicating that the division machinery is not identical between chloroplasts and proplastids. In addition, division differences were also observed in chloroplasts and chromoplasts during analysis of the tomato *suffulta* mutant [46]. Based on these reports, we speculate that the division machinery differs between the various types of plastids. In our study, we analyzed the nongreen plastids in these mutants with defects in chloroplast division site placement. Etioplasts, which differentiate from proplastids in dark-germinated seedlings [33], and previous study showed that etioplast in Zea mays could division into two daughters [47], were studied as representative of the nongreen plastids. Our data showed that normal etioplasts were present in the *arc3, arc11* and *mcd1* mutants, whereas enlarged and abnormal shaped etioplasts were observed in *parc6* and *arc12* mutants (Figure 1), suggesting that division site placement may be differently regulated in chloroplasts and nongreen plastids.

Because etioplasts are too small to be isolated, we adopted the VIGS system to detect the FtsZ-ring in nongreen plastids. The VIGS system can effect efficient and specific gene silencing, and has been widely used in reverse genetic studies in diverse plant species including tobacco, pea and Arabidopsis [30], [37], [48], [49]. Hence, we can block chloroplast development by silencing the genes involved in chloroplast biogenesis to obtain nongreen plastids. Among the proteins participating in chloroplast development, *ALB3* was reported to be involved in the assembly of a chloroplast enzyme complex, which mutated would result in a thylakoid defect [39], [40]. Consistent with the *alb3* mutant, the chloroplasts of VIGS-ALB3 plants had few thylakoid membranes and very little grana stacking (Figure 3B), but were more numerous and larger than etioplasts; thus, we can investigate the organization of FtsZ proteins by immunofluorescence using anti-FtsZ2-1 antibodies as described [23], [32]. These results here provide a new strategy to investigate the mechanisms involved in nongreen plastid division.

Our results indicate that deletion of ARC3, ARC11 and MCD1 seems not to influence the division of nongreen plastids in VIGS-ALB3 plants, while the PARC6 and ARC12 proteins were necessary (Figure 3A). In concert with this, we found that the FtsZ proteins in the three mutants in which *ALB3* had been silenced could assemble a FtsZ-ring structure at the midpoint of the nongreen plastids similar to the wild-type, to yield two daughter plastids of equal size (Figure 4). We all know that in chloroplast, the morphology of the FtsZ rings was very similar in the *arc3, arc11* and *mcd1* mutants as there were several FtsZ rings distributing in the enlarged chloroplasts. Moreover, both the MCD1 and ARC3 could interact with ARC11 to regulate the location of FtsZ proteins. Furthermore, the qRT analysis showed that the Min system genes expression pattern were similar in the light-induced etiolated seedings and VIGS-ALB3 plants (Figure S1 and S2). So we could not exclude the possibility that ARC3, ARC11 and MCD1 might function complementarily in nongreen plastid division. To test this further, double or triple mutants of Min system genes are required. However, the morphology of the FtsZ ring is much more specific in the chloroplasts of *arc12* and *parc6* mutants, suggesting that (ARC12 and PARC6) may have particular and significant function. This is possibly the reason for observing serious defect in plastid division of the etiolated seedings and VIGS-ALB3 plants lacking PARC6 or ARC12. Clearly, in our study, we show that PARC6 and ARC12 are still required for the FtsZ-ring assembly and placement in the VIGS-ALB3 plants (Figure 4) as their function in chloroplasts [16], [19]. It also has been reported that deletion of ARC12 will lead to defects in proplastid division [50].

Previous studies, together with our results, demonstrate that chloroplast division and nongreen plastid division are different. However, what causes the differences between chloroplasts and nongreen plastids? We know that, during chloroplast division, the thylakoids should also be divided between the two descendants [51]. Accordingly, we ask whether thylakoid partitioning accounts for the differences between chloroplast and nongreen plastid division. It has been reported that FtsZ proteins presented both in chloroplast stroma and thylakoid fractions, which suggests they are possibly involved in thylakoid partitioning in dividing chloroplasts [52]. In chloroplast, ARC3 interacts strongly with FtsZ1, and ARC11 interacts with MCD1 and ARC3. Therefore, the three proteins ARC3, ARC11 and MCD1 may also function in localizing the FtsZ proteins correctly on the thylakoids to make them divide properly in chloroplasts. Intriguingly, MCD1 is enriched in the chloroplast envelope fraction [21]; however, we cannot rule out the possibility that MCD1 and the other two proteins may function both in thylakoid partition and chloroplast division.

In conclusion, we show here that division site placement is differently regulated in chloroplasts and nongreen plastids (Fig. S4). Our study provides a strategy to investigate division in nongreen plastids in the future. For further study, we will focus on the functions of other proteins, such as MSL2, MSL3, etc. in nongreen plastid division and the relationship between thylakoid partitioning and chloroplast division.

## Supporting Information

Figure S1 **Expression analysis of Min system genes in etiolated seedlings.** qRT-PCR analysis of the Min system genes in the etiolated seedlings. RNA was extracted from cotyledons of 6-day-old etiolated seedlings when illuminated with light for the indicated time. Relative expression was normalized to *UBQ4* and the expression level of each gene before illumination was set to 1. Data are given as means ± SD of three biological replicates.
(PDF)

Figure S2 **Expression analysis of Min system genes in the VIGS plants.** qRT-PCR analysis of Min system genes in the VIGS plants. RNA was extracted from Col-0 two weeks after infection with TRV-GFP or TRV-ALB3. Relative gene expression was normalized to *UBQ4*, and the expression level of each gene in Col-0 (VIGS-GFP) was set to 1. Data are given as means ± SD of three biological replicates.
(PDF)

Figure S3 **Number of plastids per cell in VIGS-ALB3 plants.** Statistical comparison of the number of plastids per mesophyll cell. Blue bars show mean numbers of plastids, and error bars represent SD, n = 15.
(PDF)

Figure S4 **Working model of the coordinated division machinery in chloroplasts and nongreen plastids.** The model shows the relationships among the division components; all proteins in the division model are necessary for chloroplast division, while the proteins with grey background and yellow font may be not required for nongreen plastid division. IEM, inner envelope membrane; OEM, outer envelope membrane; IMS, intermembrane space.
(PDF)
