# Supplementary figures and images for: The Chloroplast Min System Functions Differentially in Two Specific Nongreen Plastids in Arabidopsis thaliana
Source: PLoS One. 2013 Jul 30;8(7):e71190. doi: 10.1371/journal.pone.0071190 (PMC3728212; doi:10.1371/journal.pone.0071190)

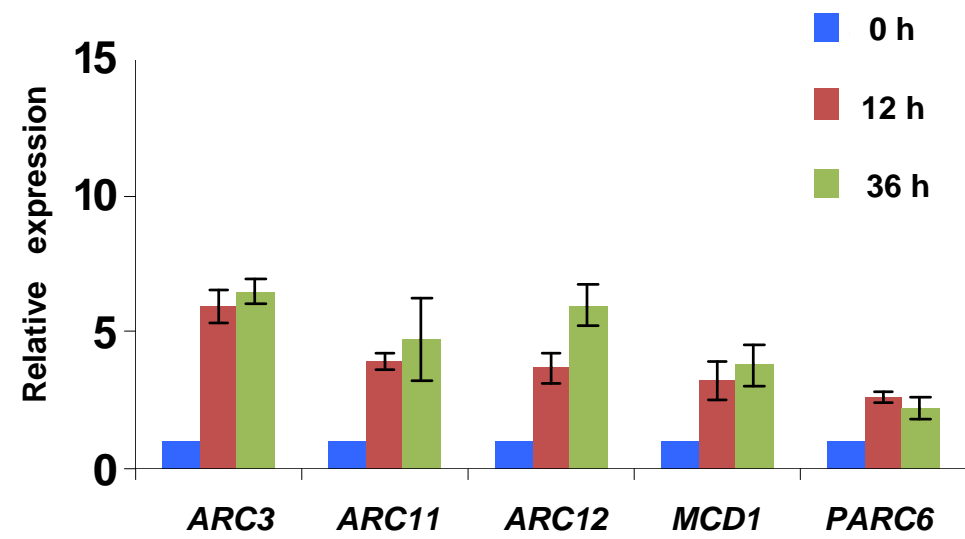

Supplement: Figure S1 — Expression analysis of Min system genes in etiolated seedlings. qRT-PCR analysis of the Min system genes in the etiolated seedlings. RNA was extracted from cotyledons of 6-day-old etiolated seedlings when illuminated with light for the indicated time. Relative expression was normalized to UBQ4 and the expression level of each gene before illumination was set to 1. Data are given as means ± SD of three biological replicates. (PDF) [file pone.0071190.s001.pdf]

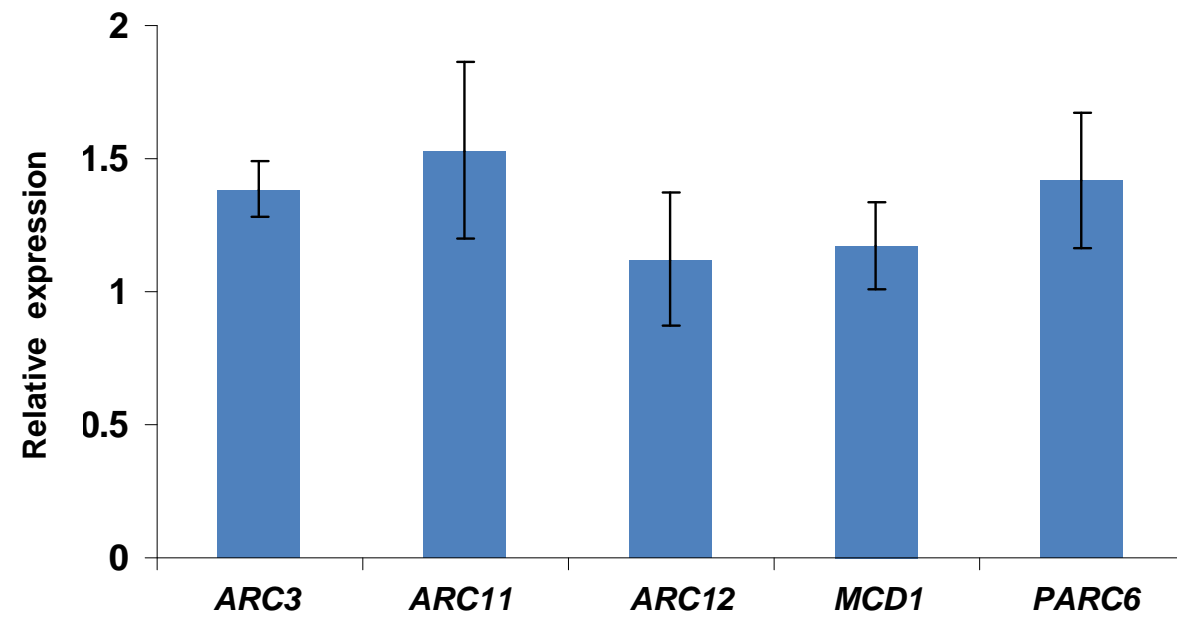

Supplement: Figure S2 — Expression analysis of Min system genes in the VIGS plants. qRT-PCR analysis of Min system genes in the VIGS plants. RNA was extracted from Col-0 two weeks after infection with TRV-GFP or TRV-ALB3. Relative gene expression was normalized to UBQ4, and the expression level of each gene in Col-0 (VIGS-GFP) was set to 1. Data are given as means ± SD of three biological replicates. (PDF) [file pone.0071190.s002.pdf]

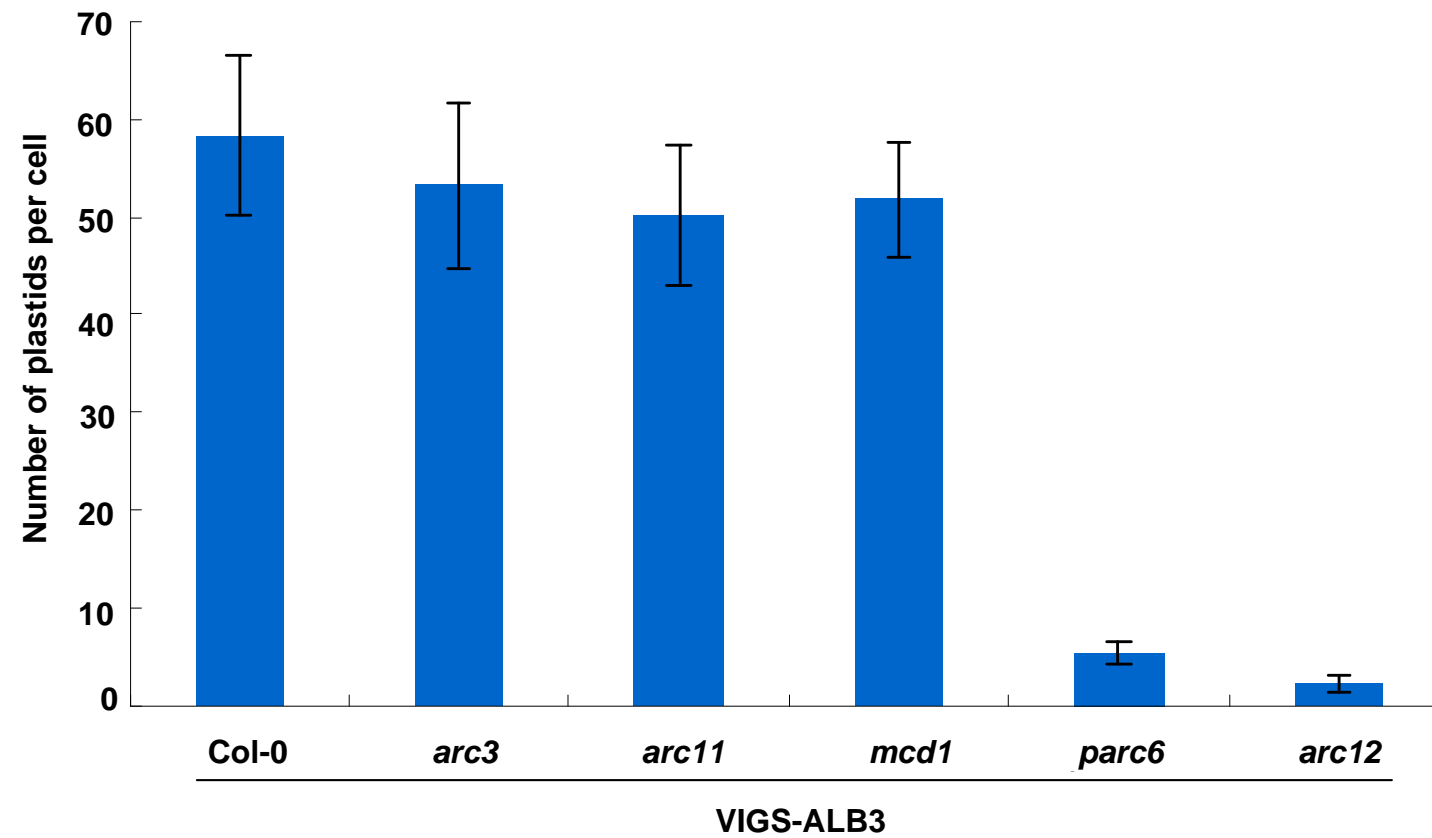

Supplement: Figure S3 — Number of plastids per cell in VIGS-ALB3 plants. Statistical comparison of the number of plastids per mesophyll cell. Blue bars show mean numbers of plastids, and error bars represent SD, n = 15. (PDF) [file pone.0071190.s003.pdf]

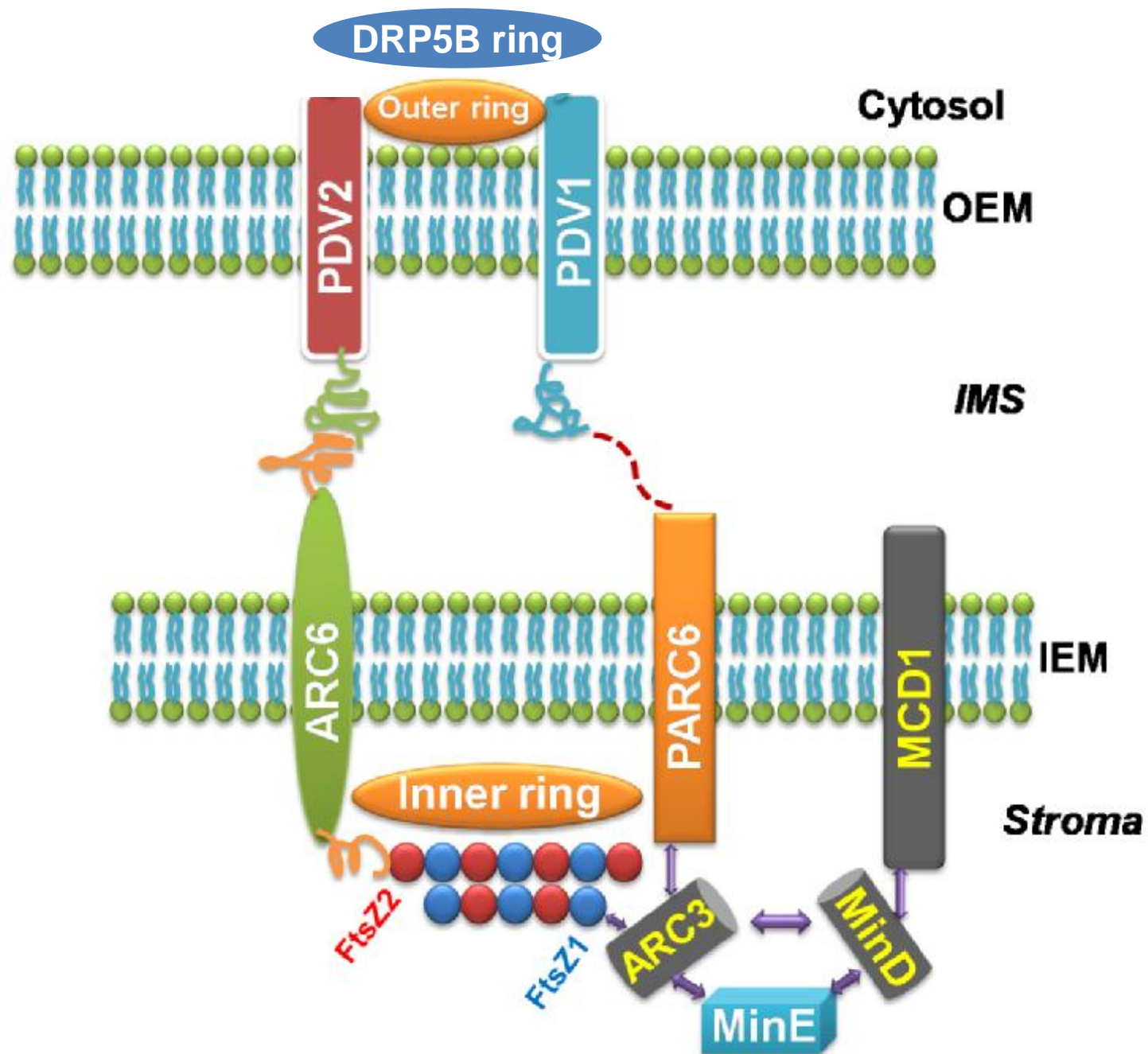

Supplement: Figure S4 — Working model of the coordinated division machinery in chloroplasts and nongreen plastids. The model shows the relationships among the division components; all proteins in the division model are necessary for chloroplast division, while the proteins with grey background and yellow font may be not required for nongreen plastid division. IEM, inner envelope membrane; OEM, outer envelope membrane; IMS, intermembrane space. (PDF) [file pone.0071190.s004.pdf]
